# Circular RNA circSP3 promotes hepatocellular carcinoma growth by sponging microRNA-198 and upregulating cyclin-dependent kinase 4

**DOI:** 10.18632/aging.203303

**Published:** 2021-07-27

**Authors:** Molin Li, Hang Chen, Lulu Xia, Ping Huang

**Affiliations:** 1 Department of Hepatobiliary Surgery, The First Affiliated Hospital of Chongqing Medical University, Chongqing Medical University, Chongqing 400000, China; 2 Department of Oncology and Hematology, The People’s Hospital of Tongliang District, Chongqing 402560, China; 3 College of Laboratory Medicine, Chongqing Medical University, Yuzhong, Chongqing 400042, China

**Keywords:** circSP3, miR-198, HCC, proliferation, migration

## Abstract

As a new class of endogenous noncoding RNAs, circular RNAs (circRNAs), have been found to influence cell development and function by sponging microRNAs. MicroRNA (miR)-198 is downregulated in various cancers, including hepatocellular carcinoma (HCC). We therefore searched for dysregulated circRNAs that could sponge miR-198 in HCC. By analyzing relevant circRNA databases (circBase, TargetScan and CircInteractome), we found that the miR-198-binding circRNA hsa_circSP3 is upregulated in HCC. CircSP3 expression correlated negatively with miR-198 expression in HCC tissues. Dual luciferase reporter assays indicated that circSP3 bound to miR-198. CircSP3 overexpression in HCC cells induced expression of cyclin-dependent kinase 4, a target gene of miR-198. Silencing circSP3 inhibited HCC cell proliferation and migration by downregulating cyclin-dependent kinase 4, whereas inhibiting miR-198 reversed those effects. *In vivo* experiments confirmed that circSP3 promoted xenograft tumor growth. These data suggest that circSP3 may be a novel biomarker for HCC.

## INTRODUCTION

Hepatocellular carcinoma (HCC) is the second most common cause of cancer-related death worldwide [1]. The high metastasis and invasiveness of HCC lead to a high recurrence rate [2], thereby resulting in a poor prognosis [3]. The pathogenesis of liver cancer involves many signaling pathways that are not fully understood, so it is imperative to further explore the molecular mechanisms of HCC to identify potential tumor markers and therapeutic targets.

Unlike linear RNAs, circular RNAs (circRNAs) are characterized by a covalent closed-loop structure with no 5' to 3' polarity and no tail polyadenylation [4, 5]. CircRNAs tend to be highly expressed in the cytoplasm of eukaryotic cells, and are conserved among species due to their resistance to RNase R [6–9]. CircRNAs can function as “super sponges” for microRNAs (miRNAs) [10, 11], and can bind to RNA binding proteins involved in tumorigenesis [12, 13]. CircRNAs broadly participate in the initiation and progression of various diseases, including malignant tumors such as gastric cancer [14, 15] and HCC [16, 17].

MiRNAs are important noncoding RNAs that inhibit the expression of protein-coding genes by binding to the 3’-untranslated regions of mRNAs [18]. Aberrant miRNA-198 (miR-198) expression has been found to correlate with the carcinogenesis and progression of various cancers, including lung adenocarcinoma [19], esophageal cancer [20], colorectal cancer [21] and prostate cancer [22]. Recent investigations have demonstrated that miR-198 is also downregulated in HCC [23]. In melanoma, the upregulation of hsa_circ_0025039 induced the downregulation of miR-198, thus promoting cancer progression by de-repressing cyclin-dependent kinase 4 (*CDK4*) [24]. Since circRNAs are known to be involved in the pathogenesis of HCC, we hypothesized that miR-198 may be downregulated in HCC due to its sponging by circRNAs.

In this study, we performed bioinformatic analyses to identify circRNAs that could bind to miR-198 in HCC, and we confirmed the results in luciferase reporter assays. We further studied one of these circRNAs, circSP3, by examining its expression in HCC tissues/cell lines and its association with tumor characteristics *in vitro* and *in vivo*.

## RESULTS

### Identification of circSP3 in HCC and confirmation of its circular structure

**Figure 1:**
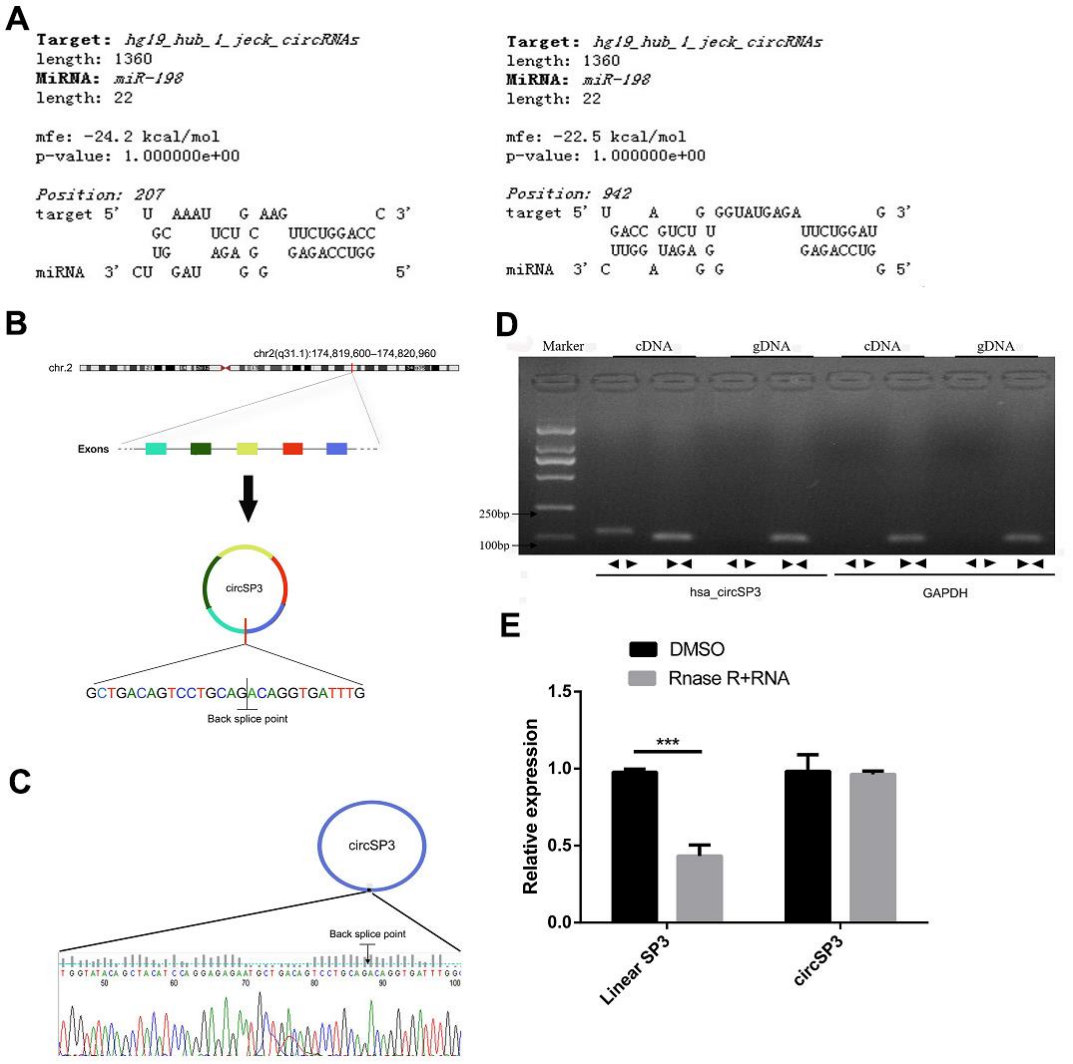
**Identification of circSP3 in HCC cells and confirmation of its circular structure.** (**A**) The predicted binding between circSP3 and miR-198. (**B**) Schematic illustration demonstrating that circSP3 is located on chromosome 2q31.1 and cyclized from exons of *SP3*. (**C**) The PCR products of circSP3 were confirmed through Sanger sequencing. (**D**) CircSP3 was detected in Huh-7 cells. As expected, divergent primers detected circSP3 in cDNA but not in gDNA. *GAPDH* was used as a negative control. (**E**) The relative levels of circSP3 and *SP3* were assessed through qRT-PCR analysis in cells treated with or without RNase R. *p<0.05, **p<0.01 and ***p<0.001.

To determine whether the downregulation of miR-198 in HCC was due to the upregulation of circRNAs, we performed a bioinformatics analysis based on the miRanda algorithm to identify circRNAs that might sponge miR-198. We found a binding site for miR-198 in circ_0002642 (circSP3) (Figure 1A). CircSP3 (chr2: 174,819,600 - 174,820,960) is derived from exons within the *SP3* locus on chromosome 2q31.1 (Figure 1B). Sanger sequencing confirmed the splice junction of circSP3 (Figure 1C).

Next, we designed two sets of primers: a divergent primer set for circular transcripts, and a convergent primer set for linear transcripts. We used these two sets of primers to amplify the circular and linear transcripts of *SP3* in both complementary DNA (cDNA) and genomic DNA (gDNA) from Huh-7 cells (an HCC cell line). The circular transcripts were amplified by divergent primers in cDNA, but not in gDNA, while the linear transcripts were amplified by convergent primers in both cDNA and gDNA. No product was amplified by divergent primers for *GAPDH* (the negative control gene) in cDNA or gDNA (Figure 1D).

The circular structure of circSP3 was confirmed in an RNase R experiment. As shown in Figure 1E, the linear transcripts of *SP3* amplified from Huh-7 cells were degraded by RNase R, while the circular transcripts of *SP3* were resistant to RNase R treatment. These data demonstrated the circular structure of circSP3.

### *CDK4* was a target of miR-198

**Figure 2:**
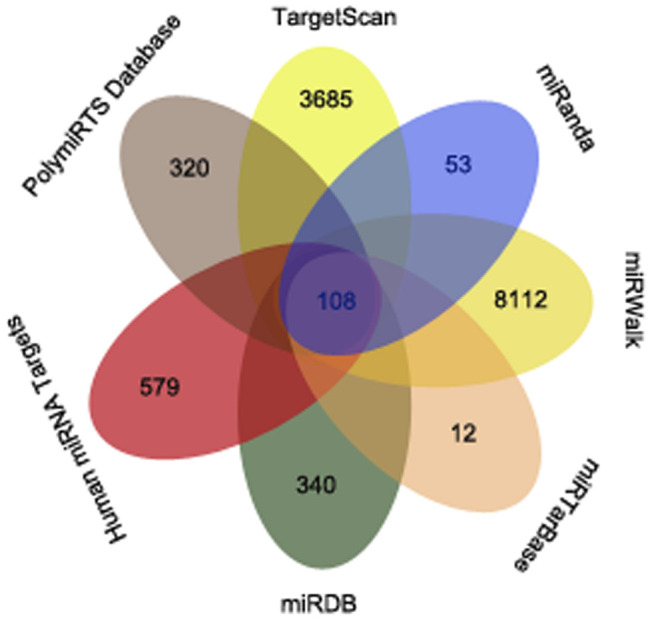
**The overlapping mRNAs from seven online analysis tools.** The mRNAs identified by TargetScan, miRanda, miRWalk, miRTarBase, miRDB, Human miRNA Targets and PolymiRTS Database are visualized with a Venn diagram.

To further investigate the involvement of miR-198 in HCC, we searched for potential miR-198 target genes in several databases, including TargetScan, miRanda, miRWalk, miRTarBase, miRDB, Human miRNA Targets and PolymiRTS Database. Only mRNAs that were predicted by four or more databases were selected for subsequent study. We further narrowed down this list by selecting genes that were specifically expressed in liver cancer, in order to identify potential miR-198 target genes in HCC. We detected 108 mRNAs to which miR-198 might bind, including *CDK4* (Figure 2). The specific mRNA names are shown in Supplementary File 1.

### CircSP3 was upregulated in HCC

**Figure 3:**
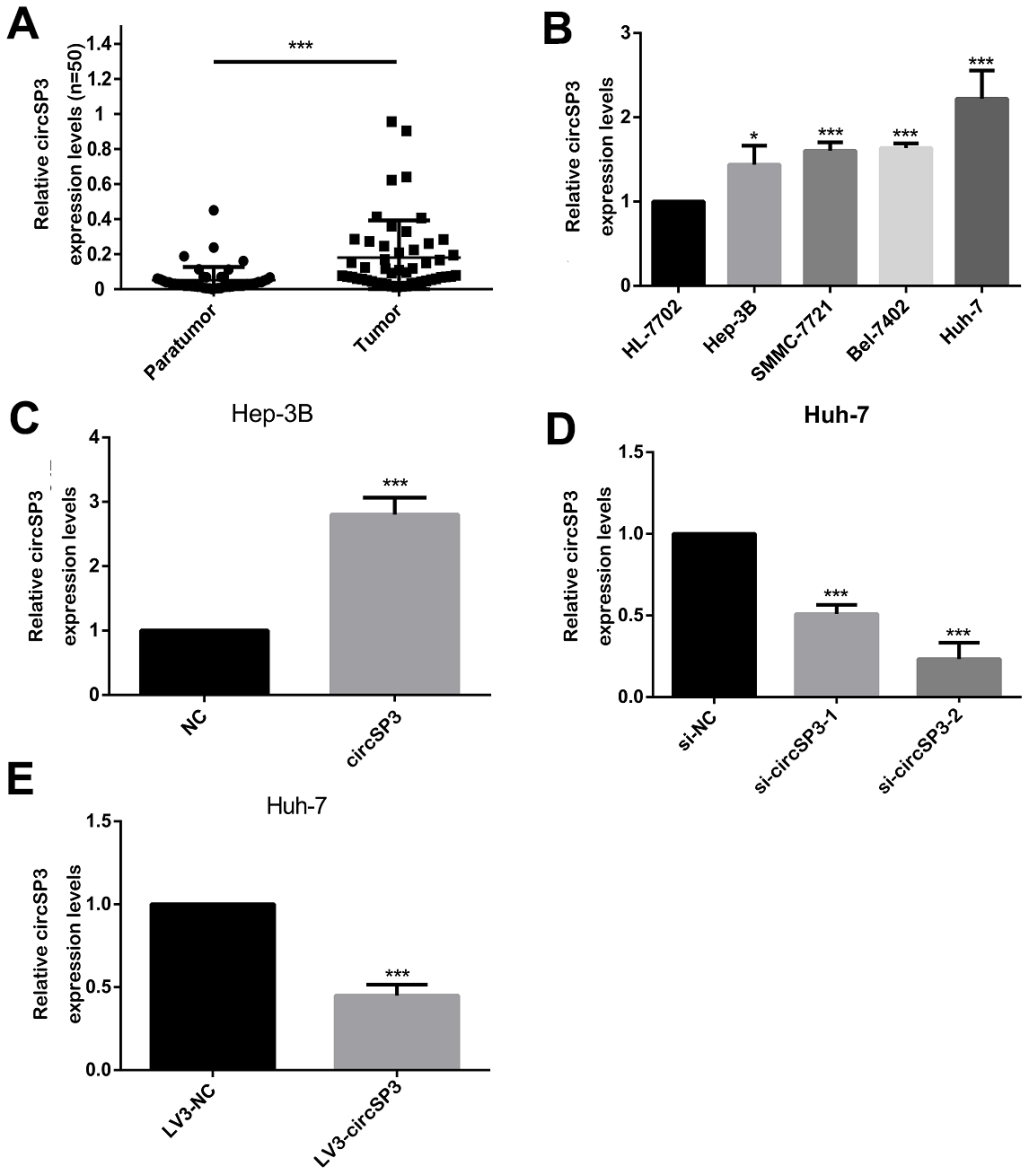
**Expression of circSP3 in HCC tissues and cell lines.** (**A**) CircSP3 levels in 48 pairs of HCC tissues and adjacent normal liver tissues were evaluated using qRT-PCR. (**B**) The relative circSP3 levels in four HCC cell lines (Hep-3B, Huh-7, Bel-7402 and SMMC-7721) and an immortalized liver cell line (HL-77O2) were determined using qRT-PCR. (**C**) CircSP3 levels in Hep-3B cells infected with NC or circSP3 plasmids. (**D**) qRT-PCR was conducted to confirm the knockdown efficiency of si-circSP3-1 and si-circSP3-2. (**E**) qRT-PCR was conducted to confirm the knockdown efficiency of LV3-circSP3. *p<0.05, **p<0.01 and ***p<0.001.

Since circSP3 was identified as a potential sponge of miR-198, we then performed quantitative real-time (qRT)-PCR to detect circSP3 levels in HCC and normal liver tissues. The results revealed that circSP3 was upregulated in HCC tissues (Figure 3A). Next, we divided patients into high and low expression groups according to the average relative circSP3 level in HCC tissues (0.1812 ± 0.02998), and we examined the clinicopathological characteristics of the two groups. We found that circSP3 levels correlated with the tumor size and tumor-node-metastasis (TNM) stage (Table 1). These results indicated that circSP3 may be a biomarker for HCC.

**Table 1:** Correlation between circSP3 expression and clinicopathological characteristics in HCC patients.

**Factor**	**Number**	**CircSP3 expression**		***x*^2^** **value**	***P* value**
		**HighN=27**	**LowN=23**		
Age				0.329	0.566
< 40 years	9	6	3		
≥ 40 years	41	21	20		
Gender				0.709	0.4
Male	41	23	18		
Female	9	4	5		
HBsAg				0.081	0.777
+	40	22	18		
-	10	5	5		
HBV DNA				1.155	0.283
+	28	17	11		
-	22	10	12		
AFP				2.498	0.114
< 400 μg/L	41	20	21		
≥ 400 μg/L	9	7	2		
ALT				0.349	0.555
< 40 U/L	26	13	13		
≥ 40 U/L	24	14	10		
AST				0.297	0.586
< 40 U/L	24	12	12		
≥ 40 U/L	26	15	11		
Tumor size				17.809	0.0001
< 5 cm	21	4	17		
≥ 5 cm	29	23	6		
TNM stage				12.268	0.002
I	14	3	11		
II	10	4	6		
III	26	20	6		

HBsAg: Hepatitis B surface antigen; HBV: Hepatitis B virus; AFP: Alpha-fetoprotein; ALT: Alanine aminotransferase; AST: Aspartate aminotransferase; TNM: Tumor-node-metastasis.

We also examined circSP3 expression in four HCC cell lines (Hep-3B, Huh-7, Bel-7402 and SMMC-7721) and a normal liver cell line (HL-77O2). CircSP3 levels were higher in the HCC cell lines than in the normal liver cell line. The lowest level was detected in Hep-3B cells, while the highest level was noted in Huh-7 cells (Figure 3B). Therefore, for subsequent experiments, Hep-3B cells were infected with circSP3-overexpressing plasmids, while Huh-7 cells were infected with small interfering RNAs (siRNAs) for circSP3 (si-circSP3-1 and si-circSP3-2) or with recombinant lentiviruses containing complementary oligonucleotides of small hairpin RNAs for circSP3 (LV3-circSP3).

We then performed qRT-PCR analyses, which demonstrated that circSP3 was significantly upregulated in Hep-3B cells that had been infected with circSP3-overexpressing plasmids (Figure 3C). On the other hand, circSP3 expression was much lower in Huh-7 cells infected with si-circSP3-2 than in those infected with the negative control (si-NC; Figure 3D). Likewise, circSP3 expression was significantly lower in Huh-7 cells infected with LV3-circSP3 than in those infected with the negative control (LV3-NC).

### CircSP3 promoted HCC cell proliferation *in vitro*

**Figure 4:**
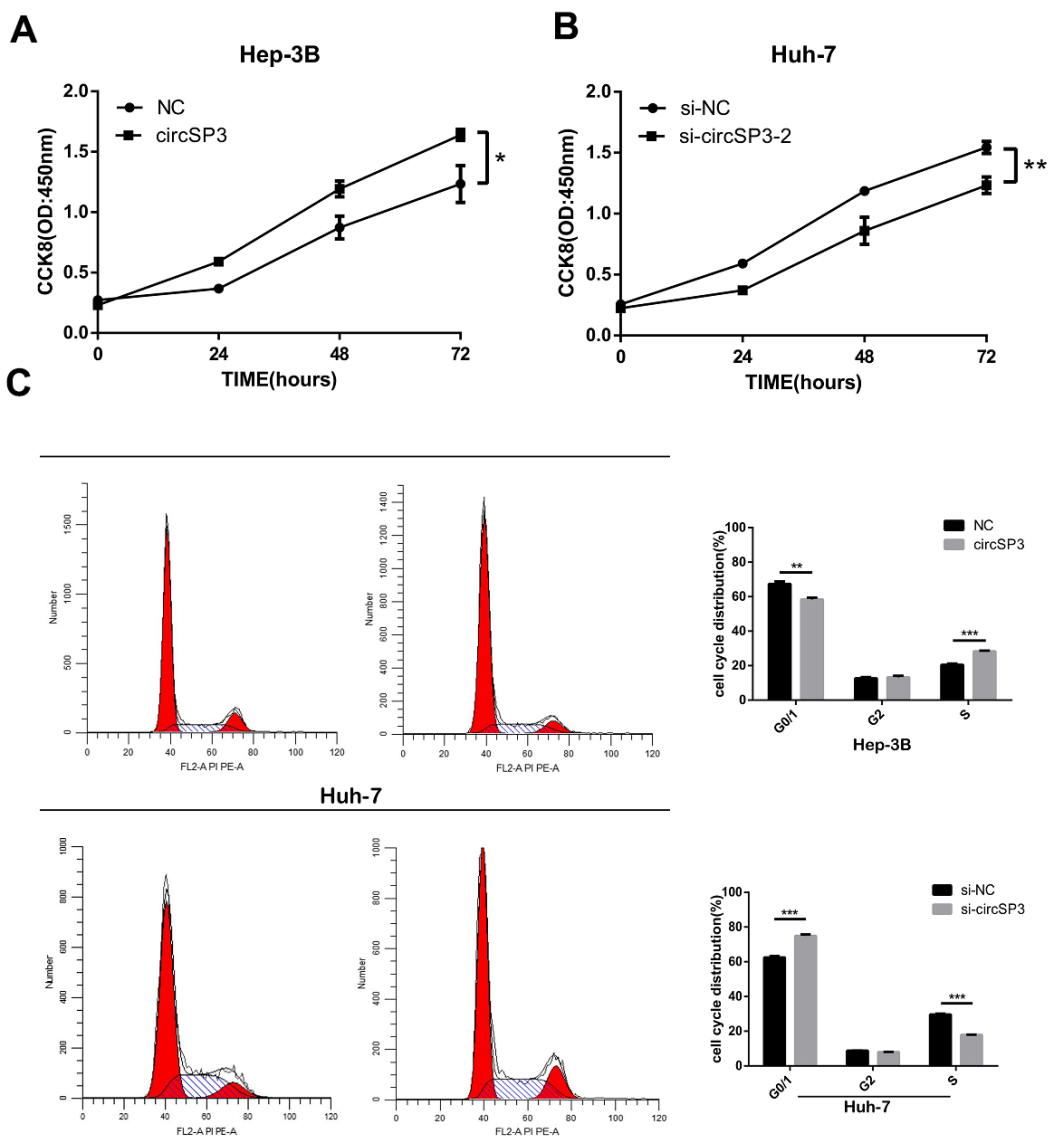
**CircSP3 induced HCC cell proliferation *in vitro*.** (**A**) A CCK-8 assay was performed to assess cell proliferation when circSP3 was overexpressed in Hep-3B cells. (**B**) A CCK-8 assay was performed to assess cell proliferation when circSP3 was knocked down in Huh-7 cells. (**C**) The cell cycle was analyzed using flow cytometry when circSP3 was overexpressed or knocked down. *p<0.05, **p<0.01 and ***p<0.001.

Next, we evaluated the effects of circSP3 overexpression or silencing on HCC cell proliferation. A Cell Counting Kit 8 (CCK-8) assay demonstrated that Hep-3B cell proliferation was enhanced when circSP3 was overexpressed (Figure 4A). In contrast, the proliferation of si-circSP3-2-infected Huh-7 cells was lower than that of si-NC-transfected cells (Figure 4B).

We also performed flow cytometry to investigate the effects of circSP3 expression on cell cycle progression and apoptosis in HCC cells. The overexpression of circSP3 significantly reduced the percentage of Hep-3B cells in G0/G1 phase and increased the percentage of Hep-3B cells in S phase. On the other hand, the knockdown of circSP3 significantly increased the percentage of Huh-7 cells in G0/G1 phase and reduced the percentage of Huh-7 cells in S phase (Figure 4C). However, circSP3 overexpression or knockdown did not alter apoptosis in HCC cells. These results indicated that circSP3 promoted HCC cell proliferation *in vitro*.

### CircSP3 promoted HCC cell migration and invasion *in vitro*

**Figure 5:**
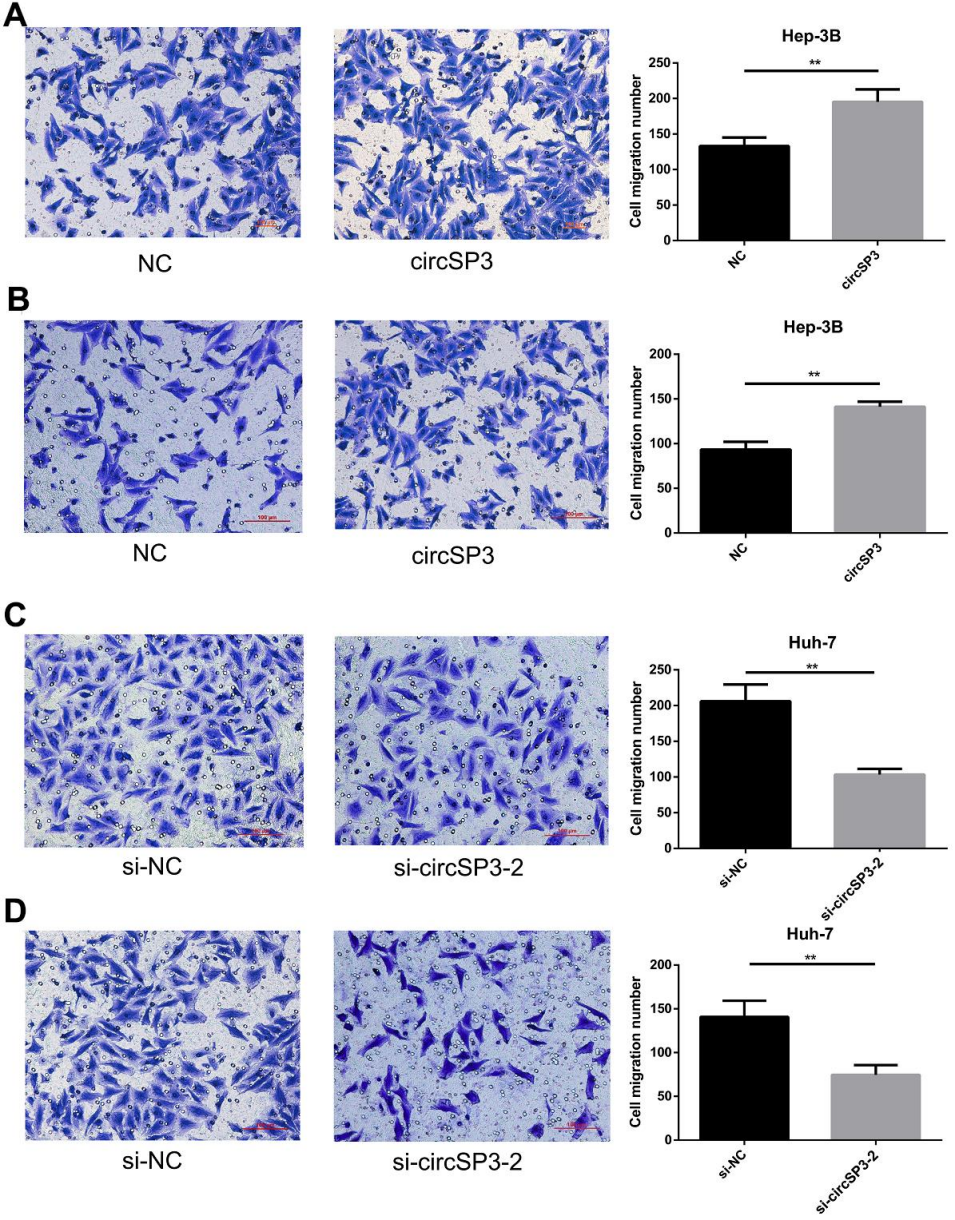
**CircSP3 induced HCC cell migration and invasion *in vitro*.** (**A**, **B**) Cell migration and invasion were assessed with Transwell assays in Hep-3B cells infected with circSP3 or NC plasmids. (**C**, **D**). Transwell assays were carried out to assess cell migration and invasion when circSP3 was downregulated in Huh-7 cells. **p<0.01.

We then assessed the influence of circSP3 overexpression or silencing on HCC cell migration and invasion. Transwell migration and invasion assays indicated that circSP3 overexpression significantly increased the migration and invasion of Hep-3B cells (Figure 5A, 5B), while si-circSP3-2 treatment reduced the migration and invasion of Huh-7 cells (Figure 5C, 5D). These results demonstrated that circSP3 promoted HCC cell migration and invasion *in vitro*.

### CircSP3 bound to miR-198 in HCC cells

**Figure 6:**
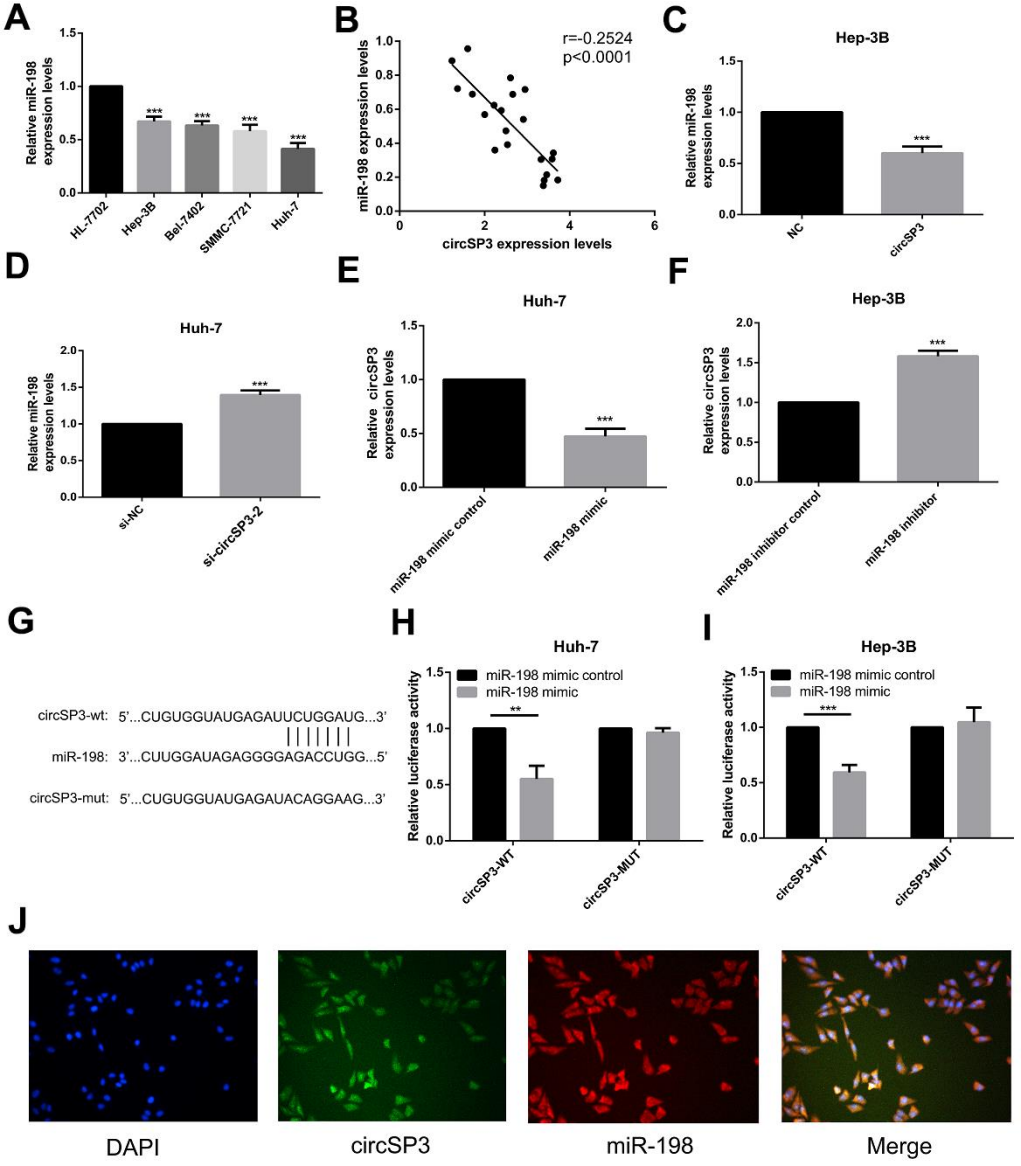
**The relationship between circSP3 and miR-198 in HCC.** (**A**) The relative miR-198 levels in four HCC cell lines (Hep-3B, Huh-7, Bel-7402 and SMMC-7721) and a normal liver cell line (HL-77O2) were assessed using qRT-PCR. (**B**) Correlation analysis between circSP3 and miR-198 levels in HCC tissues. (**C**) The relative miR-198 levels in Hep-3B cells infected with NC or circSP3 plasmids were detected using qRT-PCR. (**D**) The relative miR-198 levels in Huh-7 cells infected with si-NC or si-circSP3 plasmids were detected using qRT-PCR. (**E**) qRT-PCR revealed that ectopic expression of miR-198 reduced circSP3 expression in Huh-7 cells. (**F**) qRT-PCR demonstrated that knocking down miR-198 increased circSP3 expression in Hep-3B cells. (**G**) Schematic of predicted wild-type and mutated miR-198 binding sequences in circSP3. (**H**, **I**) Luciferase activity in HCC cells co-transfected with circSP3-wt or circSP3-mut and miR-198 mimics or miR-198 mimic controls. (**J**) FISH revealed that miR-198 colocalized with circSP3 in HCC cells. **p<0.01, ***p<0.001.

Next, we performed qRT-PCR to measure miR-198 levels in the same four HCC cell lines (Hep-3B, Huh-7, Bel-7402 and SMMC-7721) and normal liver cells (HL-77O2). The levels of miR-198 were lower in the HCC cell lines than in the normal liver cell line (Figure 6A). A correlation analysis revealed that circSP3 expression correlated negatively with miR-198 expression in HCC tissues (r=-0.2524), suggesting that sponging of miR-198 by circSP3 may promote the occurrence and development of HCC (Figure 6B).

We then performed qRT-PCR to analyze miR-198 expression in Hep-3B cells infected with circSP3-overexpressing or NC plasmids, and in Huh-7 cells infected with si-circSP3 or si-NC. The overexpression of circSP3 inhibited miR-198 expression in Hep-3B cells (Figure 6C), whereas the downregulation of circSP3 induced miR-198 expression in Huh-7 cells (Figure 6D). We also assessed circSP3 expression in Huh-7 cells transfected with miR-198 mimics or miR-198 mimic controls, and in Hep-3B cells transfected with miR-198 inhibitors or miR-198 inhibitor controls. Interestingly, the results suggested that miR-198 mimics significantly inhibited circSP3 expression (Figure 6E), whereas miR-198 inhibitors significantly promoted circSP3 expression (Figure 6F). These results indicated that circSP3 expression correlated negatively with miR-198 expression, and vice versa.

We then used a dual luciferase reporter assay to explore whether direct binding occurred between circSP3 and miR-198. The predicted binding site between miR-198 and circSP3 is shown in Figure 6G. We found that miR-198 significantly reduced the luciferase intensity of a wild-type circSP3 reporter, but had no effect on a mutated circSP3 reporter (Figure 6H, 6I). Moreover, fluorescence *in situ* hybridization (FISH) analysis revealed that circSP3 colocalized with miR-198 in the cytoplasm of HCC cells (Figure 6J). These results suggested that circSP3 could bind directly to miR-198 in the cytoplasm.

### CircSP3 promoted HCC growth by sponging miR-198 and upregulating CDK4

**Figure 7:**
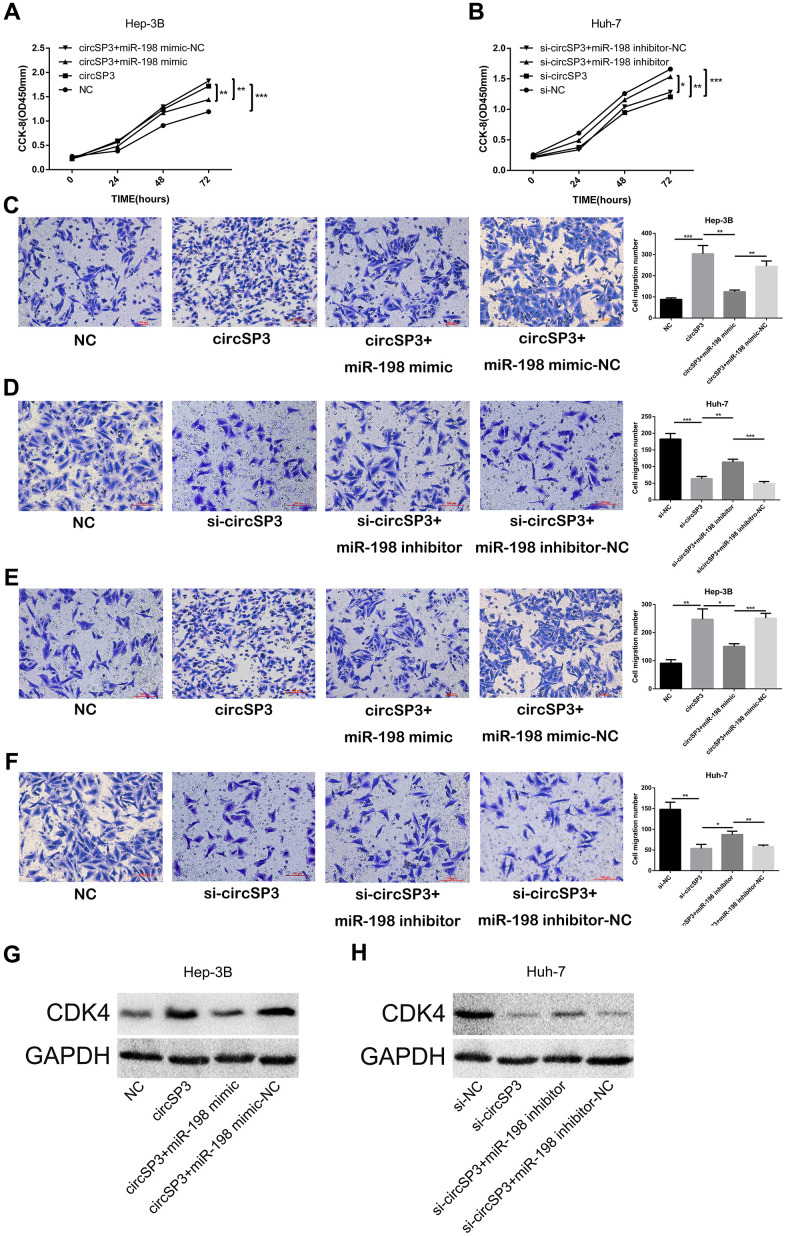
**CircSP3 induces HCC cell proliferation, migration and invasion by sponging miR-198 and upregulating CDK4.** CircSP3-overexpressing Hep-3B cells were infected with miR-198 mimics or miR-198 mimic controls, and circSP3-knockdown Huh-7 cells were infected with miR-198 inhibitors or miR-198 inhibitor controls. (**A**, **B**) CCK-8, (**C**, **D**) Transwell migration and (**E**, **F**) Transwell invasion assays were conducted to assess cell proliferation, migration and invasion, respectively. (**G**, **H**) The effects of circSP3 and miR-198 expression on CDK4 expression were quantified using Western blotting. *p<0.05, **p<0.01 and ***p<0.001.

To investigate whether circSP3 promoted tumor growth by sponging miR-198, we performed rescue experiments. Hep-3B cells were transfected with circSP3-overexpressing plasmids, NC plasmids, circSP3+miR-198 mimics or circSP3+miR-198 mimic NC plasmids. Huh-7 cells were transfected with si-circSP3, si-NC, si-circSP3+miR-198 inhibitors or si-circSP3+miR-198 inhibitor NC plasmids. CCK-8 assays and Transwell migration and invasion assays demonstrated that miR-198 overexpression reversed the enhancing effects of circSP3 on Hep-3B cell proliferation, migration and invasion (Figure 7A, 7C, 7E). On the other hand, miR-198 inhibitors abolished the inhibitory effects of si-circSP3 on Huh-7 cell proliferation, migration and invasion (Figure 7B, 7D, 7F). Notably, Western blotting indicated that circSP3 overexpression induced the protein expression of the miR-198 target CDK4, while miR-198 overexpression reversed this effect (Figure 7G). Accordingly, si-circSP3 inhibited CDK4 expression, while miR-198 inhibitors abolished this effect (Figure 7H).

**Figure 8:**
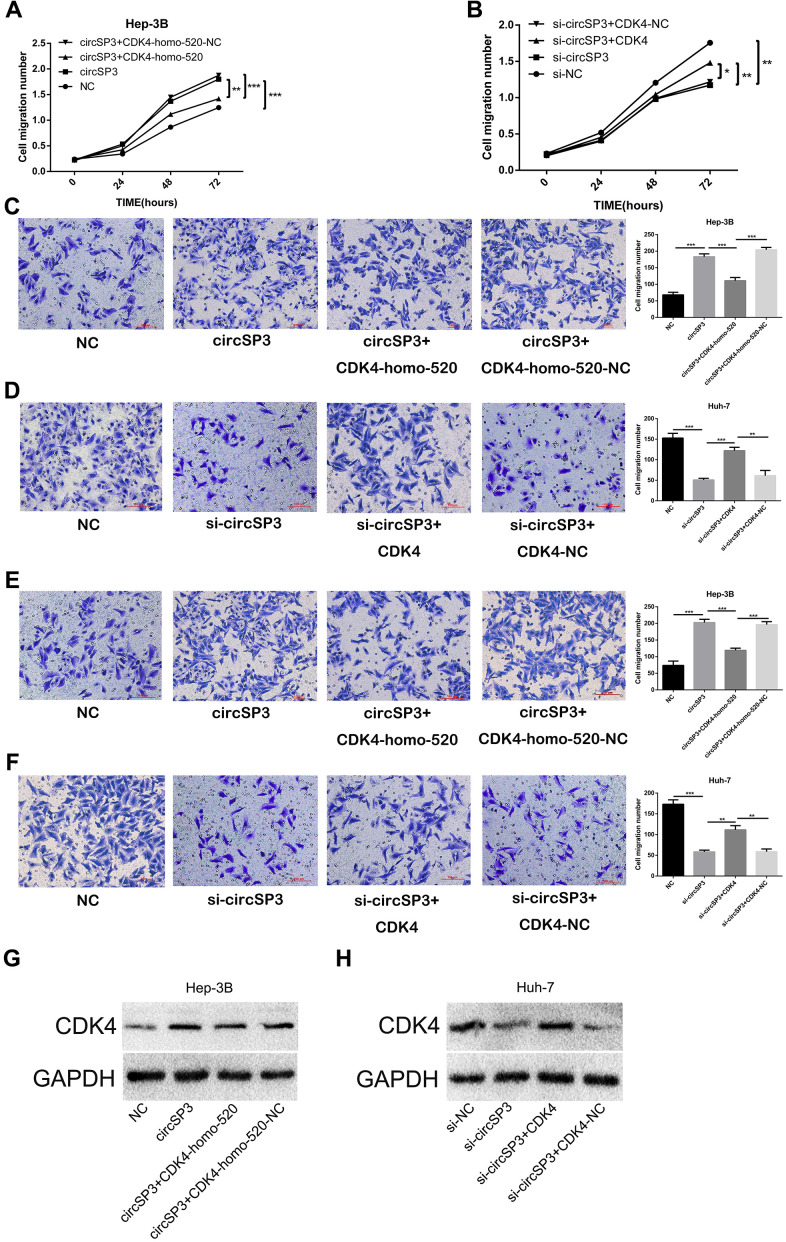
**CircSP3 promotes HCC cell proliferation, migration and invasion by inducing CDK4.** CircSP3-overexpressing Hep-3B cells were infected with CDK4-homo-520 (*CDK4* knockdown) or CDK4-homo-520 control plasmids, and circSP3-knockdown Huh-7 cells were infected with CDK4-overexpressing or CDK4 control plasmids. (**A**, **B**) CCK-8, (**C**, **D**) Transwell migration and (**E**, **F**) Transwell invasion assays were conducted to assess cell proliferation, migration and invasion, respectively. (**G**, **H**) The effects of circSP3 and CDK4 expression plasmids on CDK4 expression were quantified using Western blotting. *p<0.05, **p<0.01 and ***p<0.001.

In view of these results, we assessed whether the sponging of miR-198 by circSP3 promoted tumor growth by de-repressing *CDK4.* Hep-3B cells were transfected with circSP3-overexpressing plasmids, NC plasmids, circSP3+CDK4-homo-520 (*CDK4* knockdown) plasmids or circSP3+CDK4-homo-520-NC plasmids. Huh-7 cells were transfected with si-circSP3, si-NC, si-circSP3+CDK4 (pcDNA3.1) or si-circSP3+CDK4-NC plasmids. The inhibition of *CDK4* reversed the enhancing effects of circSP3 on Hep-3B cell proliferation, migration and invasion (Figure 8A, 8C, 8E). Furthermore, *CDK4* overexpression abolished the inhibitory effects of si-circSP3 on Huh-7 cell proliferation, migration and invasion (Figure 8B, 8D, 8F). Western blotting confirmed that knocking down *CDK4* reversed the enhancing effects of circSP3 on CDK4 expression, while overexpressing *CDK4* reversed the inhibitory effects of si-circSP3 on CDK4 expression (Figure 8G, 8H).

These data indicated that circSP3 promoted HCC cell growth by sponging miR-198 and thus inducing CDK4.

### Silencing of circSP3 inhibited HCC formation *in vivo*

**Figure 9:**
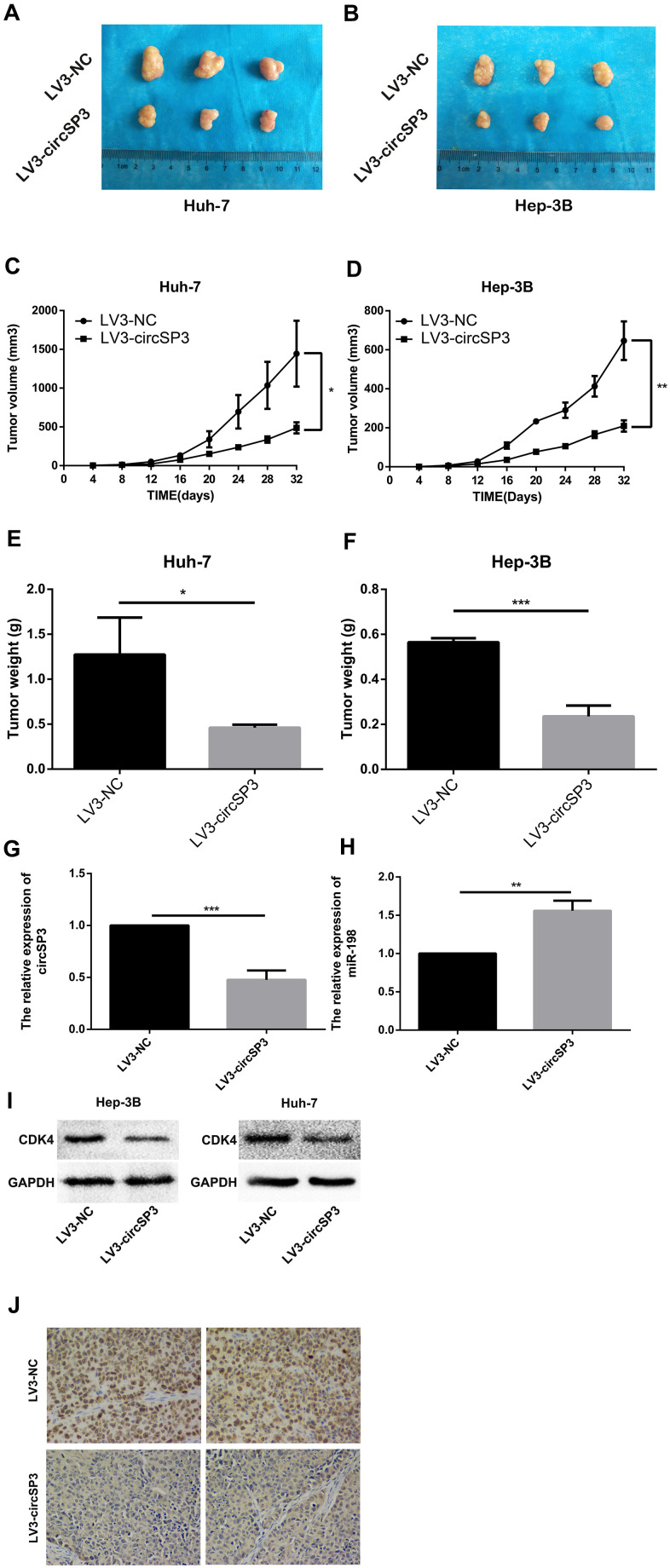
**Silencing of hsa_circSP3 inhibits HCC formation *in vivo*.** (**A**, **B**) The tumor sizes were recorded on day 32. (**C**, **D**) The rate of tumor growth was calculated every 4 days for 32 days. (**E**, **F**) The tumor weights were recorded on day 32. (**G**, **H**) The relative levels of circSP3 and miR-198 in tumors were detected using qRT-PCR. (**I**) CDK4 expression was quantified using Western blotting. (**J**) Immunohistochemistry revealed that Ki-67 levels were significantly lower in the LV3-circSP3 group than in the LV3-NC group. *p<0.05, **p<0.01 and ***p<0.001.

Finally, we investigated the effects of circSP3 on tumor growth *in vivo*. Mice were injected with Huh-7 or Hep-3B cells that had been transfected with recombinant lentiviruses containing complementary oligonucleotides of small hairpin RNAs for circSP3 (LV3-circSP3) or the negative control (LV3-NC). The tumor growth rates, volumes and weights were significantly lower in the LV3-circSP3 groups than in the LV3-NC groups (Figure 9A–9F).

We also performed qRT-PCR to detect circSP3 and miR-198, as well as Western blotting to detect CDK4, in the xenograft tumors. CircSP3 and CDK4 levels were lower in the LV3-circSP3 group than in the LV3-NC group (Figure 9G, 9I). On the other hand, miR-198 levels were higher in the LV3-circSP3 group than in the LV3-NC group (Figure 9H). In addition, we performed immunohistochemistry to detect Ki-67 levels in the xenograft tumors. Ki-67 levels were lower in the LV3- circSP3 group than in the LV3-NC group (Figure 9J). These results indicated that circSP3 enhanced tumor development *in vivo*.

## DISCUSSION

MiRNAs are important contributors to both physiological and pathological processes, including cell proliferation, apoptosis, migration and invasion [25–27]. Abnormal miRNA expression is associated with the occurrence and development of various cancers, including breast, brain, colon, liver and lung cancers and chronic myeloid leukemia [28–32]. CircRNAs, a novel class of noncoding RNAs, have also been identified as tumor biomarkers and regulators [33–35]. However, much remains unknown about the expression and function of circRNAs in HCC. In the present study, we used CircInteractome (https://circinteractome.nia.nih.gov/) and circBase (http://www.circbase.org/) to investigate whether circRNAs could bind to miR-198 in HCC, and we identified circSP3 as one such circRNA. We hypothesized that circSP3 exerts its biological functions as a sponge of miR-198 and thus an inducer of *CDK4*.

We then conducted a series of experiments to investigate the expression and function of circSP3 in HCC. CircSP3 was upregulated in HCC tissues and cell lines, and higher circSP3 expression was associated with a larger tumor size and a higher TNM stage in HCC patients. Moreover, circSP3 overexpression increased the proliferation, migration and invasion of HCC cells, while circSP3 silencing reduced these properties. Knocking down circSP3 also markedly inhibited tumor growth *in vivo*. Furthermore, qRT-PCR analysis indicated that circSP3 expression correlated negatively with miR-198 expression in HCC cells, and dual luciferase reporter assays revealed that circSP3 and miR-198 could bind directly to each other. FISH analysis demonstrated that circSP3 colocalized with miR-198 in the cytoplasm of HCC cells. In summary, we found that circSP3 exerted its biological functions as a sponge of miR-198.

We subsequently performed rescue experiments, which demonstrated that miR-198 overexpression could reverse the effects of circSP3 overexpression in HCC cells. These findings suggested that circSP3 enhanced the proliferation, migration and invasion of HCC cells by inhibiting miR-198. We also examined the effects of circSP3 and miR-198 on CDK4*,* an miR-198 target with important functions in the cell cycle and cell migration [30]. Western blotting demonstrated that circSP3 promoted CDK4 expression, while miR-198 overexpression reversed this effect. Accordingly, si-circSP3-2 inhibited CDK4 expression, while miR-198 inhibitors restored it.

In summary, our findings revealed that circSP3 was significantly upregulated in HCC and was associated with worse tumor characteristics. These data suggested that circSP3 may be a useful prognostic predictor and therapeutic target in HCC; however, more clinical data will be needed to confirm this. Our study also indicated that circSP3 could sponge miR-198 and thus induce CDK4 in HCC. Further research is needed to determine whether circSP3 also functions through other signaling pathways.

## MATERIALS AND METHODS

### Identification of circRNAs that could bind to miR-198 in HCC

We searched the CircInteractome (https://circinteractome.nia.nih.gov/) and circBase (http://www.circbase.org/) for circRNAs that could bind to miR-198 in HCC. We used strict screening conditions to predict these circRNAs, including two prediction algorithms (Pctar and miRanda), a very high stringency (>5) and a requirement for expression in at least three cancer types.

### Tissue specimens

We obtained 50 HCC tissues and matched adjacent nontumorous liver tissues (>3 cm away from the cancerous tissue) from the First Affiliated Hospital of Chongqing Medical University from August 2016 to December 2018. Patients did not receive chemotherapy or radiotherapy before surgery, and HCC was confirmed through pathological examinations. Tissues were placed in an RNA-preserving solution at 4° C overnight, and were stored at -80° C until RNA extraction. The protocol of this experiment was approved by the Local Ethics Committee and complied with the ethical guidelines of the 2013 Declaration of Helsinki.

### Cell culture

Human HCC cell lines (Hep-3B, Huh-7, Bel-7402 and SMMC-7721) and a normal liver cell line (HL-77O2) were obtained from the China Center for Type Culture Collection (Wuhan, China). Hep-3B and Huh-7 cells were cultured in Dulbecco’s modified Eagle’s medium (DMEM; Gibco, Carlsbad, CA, USA) containing 10% fetal bovine serum (PAN, Bavaria, Germany). SMMC-7721, Bel-7402 and HL-77O2 cells were cultured in RPMI1640 medium containing 10% fetal bovine serum. Cells were cultured at 37° C in 5% CO_2_.

### Cell transfection

PLVX-ZsGreen-hsa-miR-198 (miR-198 mimics), pLVX-ZsGreen (miR-198 mimic controls), pLVX-tdTomato-hsa-miR-198 inhibitor (miR-198 inhibitors) and pLVX-tdTomato (miR-198 inhibitor controls) were purchased from GeneCopoeia (Guangzhou, China). The following sequences were used: miR-198 mimics, 5'-CCG ACA ACC ACT ACC TGA-3'; miR-198 inhibitors, 5'-GAA CCU AUC UCC CCU CUG GAC C-3'; NC, 5'-CAG UAC UUU UGU GUA GUA CAA-3'.

The circSP3-overexpressing plasmids and the siRNAs targeting the junction region of circSP3 were synthesized by GenePharma (Shanghai, China). For circSP3 overexpression, the full-length coding sequence of circSP3 was cloned into the pcDNA3.1(+) vector [36]. The structure and sequence of the circSP3 overexpression plasmid are shown in Supplementary File 2. An empty pcDNA3.1(+) vector (si-NC) was used as a negative control. The siRNA sequences were: si-circSP3-1, 5'-GAC AGU CCU GCA GAC AGG UTT-3'; si-circSP3-2, 5'-GUC CUG CAG ACA GGU GAU UTT-3'; si-NC, 5'-UUC UCC GAA CGU GUC ACG UTT-3'. Hep-3B and Huh-7 cells were cultured in six-well plates. When the cells reached 50-70% confluence, they were transfected with the plasmids using Lipofectamine 2000 (Invitrogen, Carlsbad, CA, USA) according to the manufacturer’s protocol. Cells were collected for experiments after 48 h.

The lentiviral vector containing complementary oligonucleotides of small hairpin RNAs for circSP3 (LV3-circSP3) was constructed by GenePharma. An empty lentiviral vector (LV3-NC) was used as a negative control. Polybrene and puromycin were purchased from GenePharma. Cells were seeded at 1.25 x 10^6^/well in a six-well plate. When the cells reached 50-70% confluence, polybrene was used to infect them with the lentiviral vectors. Puromycin (2 μg/mL) was added to remove uninfected cells. The sequences of the vectors were: LV3-circSP3, 5'-GTC CTG CAG ACA GGT GAT T-3'; LV3-NC, 5'-TTC TCC GAA CGT GTC ACG T-3'.

The *CDK4* knockdown plasmid (CDK4-homo-520) was synthesized by GenePharma. The empty vector (CDK4-homo-520-NC) was used as a negative control. The sequences of the plasmids were: CDK4-homo-520, 5'-GCC AGU UUC UAA GAG GCC UTT-3'; CDK4-homo-520-NC, 5'-UUC UCC GAA CGU GUC ACG UTT-3'.

### RNA extraction, qRT-PCR and RNase R treatment

Total RNA was extracted with TRIzol reagent (Invitrogen). The extracted RNA was digested with RNase R (20 U/μL, Epicentre, USA) to remove linear RNAs and enrich circRNAs. Reverse transcription and qRT-PCR were performed with a circular RNA fluorescent quantitative detection kit (GENESEED, Guangzhou, China). The reverse transcription reaction system for circRNA was as follows:

**Table:** 

**Reagent**	**volume**
Total RNA	2 μL
2×RT Buffer	10 μL
TransScript RT Enzyme (M-MLV) Reverse Transcription Primer	1 μL 2 μL
ddH2O	To 20 μL

The reverse transcription reaction conditions included incubation at 25° C for 10 min, 42° C for 15 min, 85° C for 5 min and 4° C for storage.

The qRT-PCR reaction system was as follows:

**Table:** 

**Reagent**	**volume**
2× qPCR SYBR Green Master Mix	10 μL
Forward primer (10 μM)	0.5 μL
Reverse primer (10 μM)	0.5 μL
cDNA	1 μL
ddH2O	8 μL

The qRT-PCR reaction conditions included pre-denaturation at 95° C for 5 min, followed by 40 cycles of 95° C for 15 s, 60° C for 15 s and 72° C for 32 s.

A CFX96 Real-Time PCR System (Bio-Rad, USA) was used for qRT-PCR. The following primers were used: *GAPDH*, 5′-GTA TGA CAA CGA ATT TGG CTA CAG-3′ (forward), 5′-TGA GGG TCT CTC TCT TCC TCT TGT- 3′ (reverse); circSP3, 5′-CTC CAG TTA GTC TAA GCA CTG G-3′ (forward), 5′-GCC AAA TCA CCT GTC TGC AG-3′ (reverse); *SP3*, 5′-GAC AAT GAC TGC AGG CAT TAA T-3′ (forward), 5′-TAA TAT CAG GAG AAA CCC GCT C-3′ (reverse); miR-198, 5′-ATA TGT CAC TAG GTC CAG AGG GG -3′ (forward), 5′-TAT GGT TGT TCT GCT CTC TGT GTC -3′ (reverse); *U6*, 5′-CTC GCT TCG GCA GCA CA-3′ (forward), 5′-AAC GCT TCA CGA ATT TGC GT-3′ (reverse).

### Circular structure confirmation

The circular structure of circSP3 was confirmed through Sanger sequencing and divergent primer PCR treatment. PCR products amplified by divergent primers for circSP3 were inserted into a T vector and delivered to Tsingke Biotech for Sanger sequencing. The results were crosschecked with the back-spliced region of circSP3 supplied by circBase. In addition, since circRNAs normally arise from pre-mRNAs, divergent and convergent primers were used to amplify the circular and linear transcripts of *SP3*, respectively, in both cDNA and gDNA from Huh-7 cells. Theoretically, the circular transcript of *SP3* could be only amplified by divergent primers in cDNA, not gDNA. *GAPDH* was employed as the negative control. The primer sequences were: human *GAPDH*, GCC GTC TAG AAA AAC CTG CC (forward), CCA CCT GGT GCT CAG TGT AG (reverse); circ-*GAPDH*, GTG CTC AAC CAG TTA GCT CTC (forward), CCA AAT CCG TTG ACT CCG AC (reverse); hsa_circ_SP3, CTC CAG TTA GTC TAA GCA CTG G (forward), AAG CCA AAT CAC CTG TCT GCA (reverse); *SP3*, CAT CTG CGT TGG CAT TCT GG (forward), ATG TGT TCT TCT GTG CCT CTG T (reverse).

### CCK-8 assay

Cell proliferation was assessed with a CCK-8 assay (Hanbio Biotechnology Co. Ltd., Shanghai, China). Cells (2 × 10^3^) were seeded into 96-well plates. At six different time points, 10 μL of CCK-8 solution was added to each well. After 2 h of incubation at 37° C, the absorbance at 450 nm was measured on a microplate reader (Thermo Scientific, USA). Experiments were independently performed in triplicate.

### Transwell migration and invasion assays

Cell migration and invasion were assessed using Transwell chambers (8.0 μm pore size; EMD Millipore, Billerica, MA, USA) with or without Matrigel (diluted 1:9) (Corning Inc., USA), respectively. For the migration assay, infected Hep-3B cells (4×10^5^) or Huh-7 cells (2×10^5^) were resuspended in 200 μL of serum-free MEM (Minimum Essential Medium) and seeded into the upper chamber, while 500 μL of DMEM containing 10% serum was added to the lower chamber. For the invasion assay, cell-containing medium was seeded into the Matrigel-coated upper chamber, and 500 μL of DMEM containing 10% serum was added to the lower chamber. After 24 h, the cells in the upper chamber were removed with cotton swabs, while those on the lower membrane were fixed in 4% paraformaldehyde for 30 min and then stained with 0.1% crystal violet (Beyotime, Jiangsu, China) for 30 min. Cells were counted under an upright microscope (Nikon, Tokyo, Japan, 200× magnification).

### Cell cycle and apoptosis assay

To assess the cell cycle and apoptosis, we seeded 3 × 10^5^ treated cells into six-well plates and incubated them at 37° C for 48 h. For the cell cycle analysis, cells were digested with trypsin (Hyclone), washed twice with phosphate-buffered saline (PBS) and fixed in 70% ethanol at 4° C overnight. The cells were centrifuged at 500 x *g* for 5 min, washed twice with cold PBS and centrifuged. Cell cycle analysis was performed using fluorescence-activated cell sorting after the digested cells were treated with RNase A (0.1 mg/mL) and stained with propidium iodide (0.05 mg/mL; 4A Biotech, Beijing, China) for 30 min at 37° C.

For the apoptosis analysis, cells were digested with trypsin and washed twice with PBS. The cells were then stained for 5 min at room temperature using an Annexin V/propidium iodide detection kit (4A Biotech). Apoptotic cells were measured using flow cytometry (Beckman Coulter). All experiments were repeated at least three times.

### Luciferase reporter assay

The pmiRGLO-circSP3-wildtype (circSP3-wt) or miRGLO-circSP3-mutant (circSP3-mut) reporter vectors containing the predicted miR-198 binding sites were purchased from GenePharma. The circSP3-wt or circSP3-mut vectors were co-transfected with miR-198 mimics or miR-198 mimic controls into cells using Lipofectamine 2000 (Invitrogen). After 48 h, firefly and Renilla luciferase activities were measured with a Dual Luciferase Reporter Assay system (Promega, USA). Firefly luciferase activity was normalized to Renilla luciferase activity.

### Protein extraction and western blot analysis

Radioimmunoprecipitation assay lysis buffer was used for cell lysis and total protein extraction. The total protein content was quantified with a bicinchoninic acid protein assay kit (Thermo Fisher Scientific). The protein extracts were combined with protein loading buffer and boiled for 10 min. Then, 15 μg of each protein sample was separated on a 10% sodium dodecyl sulfate polyacrylamide gel (80 V for 40 min, 120 V for 80 min) (Beyotime) and transferred to a polyvinylidene difluoride membrane (210 mA constant current, 60 min) (Millipore). The membrane was blocked with 5% skimmed milk at room temperature for 2 h and then incubated with anti-CDK4 (1:1000, Cell Signaling Technology, USA, 12790s) or anti-GAPDH (1:500, Bioss, China, bs-0755R) primary antibodies overnight at 4° C. The membranes were subsequently incubated with goat anti-rabbit IgG secondary antibodies conjugated to horseradish peroxidase (1:5000, Bioss, bs-0295G-HRP) at room temperature for 2 h. Finally, the proteins were visualized with a Western Bright ECL Kit (Advansta, USA).

### RNA fish

Cell suspensions were pipetted onto autoclaved slides in 48-well plates, washed with PBS and fixed with 4% paraformaldehyde. After dehydration was performed with 70, 85 and 100% ethanol, hybridization was conducted overnight with circSP3 and hsa-miR-198 probes. Treatment was performed using a FISH kit (GenePharma). Then, 100 μL of diluted 4′,6-diamidino-2-phenylindole stain was added to each well for 20 min in the dark. The staining solution was then discarded, 100 μL of PBS was added, and the coverslips were removed and observed under a fluorescence microscope. The sequence of the circSP3 probe was 5′-AAA AGC CCG TGA AAC AAG AGG AAG CCC GTG AAA CAA CAG GAA ATG GAA ATG GCT GCC TTG GAC GTG GAT-3′, and the sequence of the hsa-miR-198 probe was 5′-GAA CCT ATC TCC CCT CTG GAC C-3′.

### HCC xenograft mouse model

Four-week-old female BALB/c nude mice were obtained from the Laboratory Animal Center of Chongqing Medical University (Chongqing, China). The hip of each mouse was subcutaneously injected with 5×10^6^ Huh-7 or Hep-3B cells infected with LV3-circSP3 or LV3-NC recombinant lentiviruses (n=3 mice/group). The tumor volumes were measured every four days (length × width^2^ / 2) (mm^3^). All mice were sacrificed on day 32, and their tumors were removed, weighed and photographed.

### Immunohistochemistry assay

Dissected tumor tissues were fixed overnight with a formalin solution, dehydrated with ethanol, embedded in paraffin and sectioned at 5 mm. The slides were blocked with 5% normal goat serum and then incubated with anti-Ki-67 antibodies (Bioss, bs-23103R) at 4° C overnight. After being washed, the slides were incubated with goat anti-rabbit horseradish peroxidase antibodies (Vector Laboratories). The proteins were visualized with 3,3′-diaminobenzidine (Sigma-Aldrich, St. Louis, MO, USA) and analyzed under a light microscope (Nikon). The staining intensity was graded on the following scale: 0 (absence of staining), 1 (weakly stained), 2 (moderately stained) and 3 (strongly stained). The percentage of positive tumor cells was scored as follows: 0 (absence of tumor cells), 1 (<33% tumor cells), 2 (33-66% tumor cells) and 3 (>66% tumor cells).

### Statistical analysis

All statistical analyses were performed using GraphPad software 6.0 (GraphPad Inc., San Diego, CA, USA) and SPSS version 24.0 (Chicago, IL, USA). Each experiment was repeated at least three times. Data are presented as the mean ± standard deviation. Differences were considered statistically significant at p<0.05.

## Supplementary Material

Supplementary Files

## References

[1] Ferlay J, Soerjomataram I, Dikshit R, Eser S, Mathers C, Rebelo M, Parkin DM, Forman D, Bray F. Cancer incidence and mortality worldwide: sources, methods and major patterns in GLOBOCAN 2012. Int J Cancer. 2015; 136:E359–86. 10.1002/ijc.29210 25220842

[2] Forner A, Llovet JM, Bruix J. Hepatocellular carcinoma. Lancet. 2012; 379:1245–55. 10.1016/S0140-6736(11)61347-0 22353262 PMC13537732

[3] Ghouri YA, Mian I, Rowe JH. Review of hepatocellular carcinoma: Epidemiology, etiology, and carcinogenesis. J Carcinog. 2017; 16:1. 10.4103/jcar.JCar_9_16 28694740 PMC5490340

[4] Llovet JM, Zucman-Rossi J, Pikarsky E, Sangro B, Schwartz M, Sherman M, Gores G. Hepatocellular carcinoma. Nat Rev Dis Primers. 2016; 2:16018. 10.1038/nrdp.2016.18 27158749

[5] Torre LA, Bray F, Siegel RL, Ferlay J, Lortet-Tieulent J, Jemal A. Global cancer statistics, 2012. CA Cancer J Clin. 2015; 65:87–108. 10.3322/caac.21262 25651787

[6] Qu S, Zhong Y, Shang R, Zhang X, Song W, Kjems J, Li H. The emerging landscape of circular RNA in life processes. RNA Biol. 2017; 14:992–99. 10.1080/15476286.2016.1220473 27617908 PMC5680710

[7] Qu S, Yang X, Li X, Wang J, Gao Y, Shang R, Sun W, Dou K, Li H. Circular RNA: A new star of noncoding RNAs. Cancer Lett. 2015; 365:141–48. 10.1016/j.canlet.2015.06.003 26052092

[8] Hansen TB, Jensen TI, Clausen BH, Bramsen JB, Finsen B, Damgaard CK, Kjems J. Natural RNA circles function as efficient microRNA sponges. Nature. 2013; 495:384–88. 10.1038/nature11993 23446346

[9] Jeck WR, Sorrentino JA, Wang K, Slevin MK, Burd CE, Liu J, Marzluff WF, Sharpless NE. Circular RNAs are abundant, conserved, and associated with ALU repeats. RNA. 2013; 19:141–57. 10.1261/rna.035667.112 23249747 PMC3543092

[10] Zheng F, Liao YJ, Cai MY, Liu YH, Liu TH, Chen SP, Bian XW, Guan XY, Lin MC, Zeng YX, Kung HF, Xie D. The putative tumour suppressor microRNA-124 modulates hepatocellular carcinoma cell aggressiveness by repressing ROCK2 and EZH2. Gut. 2012; 61:278–89. 10.1136/gut.2011.239145 21672940

[11] Sanger HL, Klotz G, Riesner D, Gross HJ, Kleinschmidt AK. Viroids are single-stranded covalently closed circular RNA molecules existing as highly base-paired rod-like structures. Proc Natl Acad Sci USA. 1976; 73:3852–56. 10.1073/pnas.73.11.3852 1069269 PMC431239

[12] Suzuki H, Tsukahara T. A view of pre-mRNA splicing from RNase R resistant RNAs. Int J Mol Sci. 2014; 15:9331–42. 10.3390/ijms15069331 24865493 PMC4100097

[13] Hansen TB, Kjems J, Damgaard CK. Circular RNA and miR-7 in cancer. Cancer Res. 2013; 73:5609–12. 10.1158/0008-5472.CAN-13-1568 24014594

[14] Chen J, Li Y, Zheng Q, Bao C, He J, Chen B, Lyu D, Zheng B, Xu Y, Long Z, Zhou Y, Zhu H, Wang Y, et al. Circular RNA profile identifies circPVT1 as a proliferative factor and prognostic marker in gastric cancer. Cancer Lett. 2017; 388:208–19. 10.1016/j.canlet.2016.12.006 27986464

[15] Zhang J, Liu H, Hou L, Wang G, Zhang R, Huang Y, Chen X, Zhu J. Circular RNA_LARP4 inhibits cell proliferation and invasion of gastric cancer by sponging miR-424-5p and regulating LATS1 expression. Mol Cancer. 2017; 16:151. 10.1186/s12943-017-0719-3 28893265 PMC5594516

[16] Han D, Li J, Wang H, Su X, Hou J, Gu Y, Qian C, Lin Y, Liu X, Huang M, Li N, Zhou W, Yu Y, Cao X. Circular RNA circMTO1 acts as the sponge of microRNA-9 to suppress hepatocellular carcinoma progression. Hepatology. 2017; 66:1151–64. 10.1002/hep.29270 28520103

[17] Yu J, Xu QG, Wang ZG, Yang Y, Zhang L, Ma JZ, Sun SH, Yang F, Zhou WP. Circular RNA cSMARCA5 inhibits growth and metastasis in hepatocellular carcinoma. J Hepatol. 2018; 68:1214–27. 10.1016/j.jhep.2018.01.012 29378234

[18] Hatziapostolou M, Polytarchou C, Aggelidou E, Drakaki A, Poultsides GA, Jaeger SA, Ogata H, Karin M, Struhl K, Hadzopoulou-Cladaras M, Iliopoulos D. An HNF4α-miRNA inflammatory feedback circuit regulates hepatocellular oncogenesis. Cell. 2011; 147:1233–47. 10.1016/j.cell.2011.10.043 22153071 PMC3251960

[19] Han HS, Yun J, Lim SN, Han JH, Lee KH, Kim ST, Kang MH, Son SM, Lee YM, Choi SY, Yun SJ, Kim WJ, Lee OJ. Downregulation of cell-free miR-198 as a diagnostic biomarker for lung adenocarcinoma-associated malignant pleural effusion. Int J Cancer. 2013; 133:645–52. 10.1002/ijc.28054 23354517

[20] Qi B, Yao WJ, Zhao BS, Qin XG, Wang Y, Wang WJ, Wang TY, Liu SG, Li HC. Involvement of microRNA-198 overexpression in the poor prognosis of esophageal cancer. Asian Pac J Cancer Prev. 2013; 14:5073–76. 10.7314/apjcp.2013.14.9.5073 24175778

[21] Wang M, Wang J, Kong X, Chen H, Wang Y, Qin M, Lin Y, Chen H, Xu J, Hong J, Chen YX, Zou W, Fang JY. MiR-198 represses tumor growth and metastasis in colorectal cancer by targeting fucosyl transferase 8. Sci Rep. 2014; 4:6145. 10.1038/srep06145 25174450 PMC5385833

[22] Walter BA, Valera VA, Pinto PA, Merino MJ. Comprehensive microRNA Profiling of Prostate Cancer. J Cancer. 2013; 4:350–57. 10.7150/jca.6394 23781281 PMC3677622

[23] Huang WT, Wang HL, Yang H, Ren FH, Luo YH, Huang CQ, Liang YY, Liang HW, Chen G, Dang YW. Lower expressed miR-198 and its potential targets in hepatocellular carcinoma: a clinicopathological and in silico study. Onco Targets Ther. 2016; 9:5163–80. 10.2147/OTT.S108828 27578984 PMC5001667

[24] Bian D, Wu Y, Song G. Novel circular RNA, hsa_circ_0025039 promotes cell growth, invasion and glucose metabolism in malignant melanoma via the miR-198/CDK4 axis. Biomed Pharmacother. 2018; 108:165–76. 10.1016/j.biopha.2018.08.152 30219673

[25] Armand-Labit V, Pradines A. Circulating cell-free microRNAs as clinical cancer biomarkers. Biomol Concepts. 2017; 8:61–81. 10.1515/bmc-2017-0002 28448269

[26] Ji W, Sun B, Su C. Targeting MicroRNAs in Cancer Gene Therapy. Genes (Basel). 2017; 8:21. 10.3390/genes8010021 28075356 PMC5295016

[27] Vannini I, Fanini F, Fabbri M. Emerging roles of microRNAs in cancer. Curr Opin Genet Dev. 2018; 48:128–33. 10.1016/j.gde.2018.01.001 29429825 PMC5986298

[28] Wu Q, Yang Z, Shi Y, Fan D. MiRNAs in human cancers: the diagnostic and therapeutic implications. Curr Pharm Des. 2014; 20:5336–47. 10.2174/1381612820666140128204914 24479806

[29] Castro D, Moreira M, Gouveia AM, Pozza DH, De Mello RA. MicroRNAs in lung cancer. Oncotarget. 2017; 8:81679–85. 10.18632/oncotarget.20955 29113423 PMC5655318

[30] Enokida H, Yoshino H, Matsushita R, Nakagawa M. The role of microRNAs in bladder cancer. Investig Clin Urol. 2016 (Suppl 1); 57:S60–76. 10.4111/icu.2016.57.S1.S60 27326409 PMC4910767

[31] Flatmark K, Høye E, Fromm B. microRNAs as cancer biomarkers. Scand J Clin Lab Invest Suppl. 2016; 245:S80–83. 10.1080/00365513.2016.1210330 27542003

[32] Kim SW. [The Role of MicroRNAs in Colorectal Cancer]. Korean J Gastroenterol. 2017; 69:206–11. 10.4166/kjg.2017.69.4.206 28449421

[33] Li Y, Zheng Q, Bao C, Li S, Guo W, Zhao J, Chen D, Gu J, He X, Huang S. Circular RNA is enriched and stable in exosomes: a promising biomarker for cancer diagnosis. Cell Res. 2015; 25:981–84. 10.1038/cr.2015.82 26138677 PMC4528056

[34] Memczak S, Papavasileiou P, Peters O, Rajewsky N. Identification and Characterization of Circular RNAs As a New Class of Putative Biomarkers in Human Blood. PLoS One. 2015; 10:e0141214. 10.1371/journal.pone.0141214 26485708 PMC4617279

[35] Qin M, Liu G, Huo X, Tao X, Sun X, Ge Z, Yang J, Fan J, Liu L, Qin W. Hsa_circ_0001649: A circular RNA and potential novel biomarker for hepatocellular carcinoma. Cancer Biomark. 2016; 16:161–69. 10.3233/CBM-150552 26600397 PMC13016540

[36] Wesselhoeft RA, Kowalski PS, Anderson DG. Engineering circular RNA for potent and stable translation in eukaryotic cells. Nat Commun. 2018; 9:2629. 10.1038/s41467-018-05096-6 29980667 PMC6035260

