# Supplementary material for: Circular RNA circSP3 promotes hepatocellular carcinoma growth by sponging microRNA-198 and upregulating cyclin-dependent kinase 4
Source: Aging (Albany NY). 2021 Jul 27;13(14):18586–605. doi: 10.18632/aging.203303 (PMC8351711; doi:10.18632/aging.203303)
Supplement: Supplementary Files [file aging-13-203303-s001.pdf]

SUPPLEMENTARY FILES

Supplementary File 1.-circSP3 overexpression plasmid structure and sequence.

The structure of circSP3 overexpression plasmid is as follows:

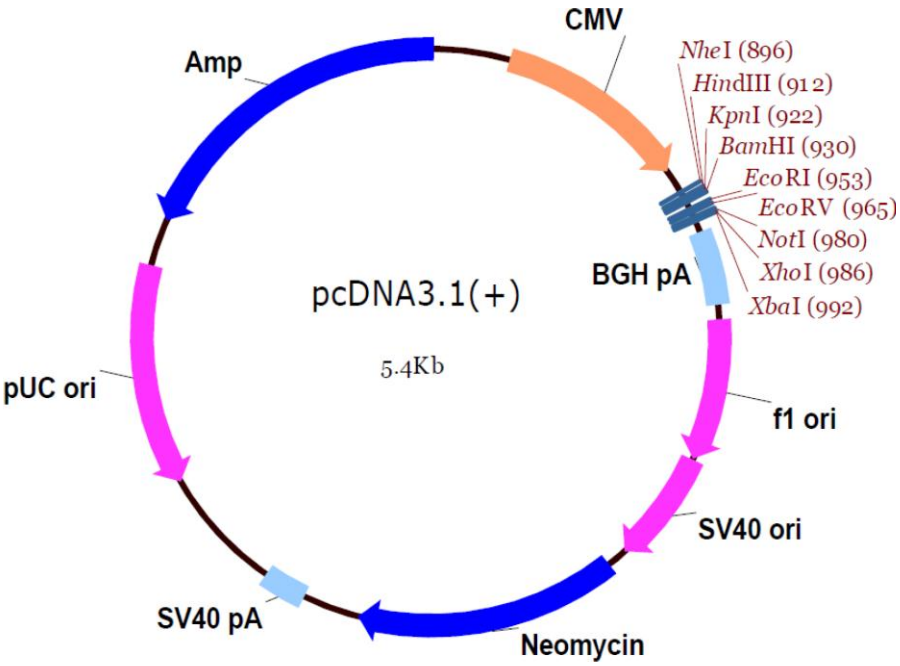

Cloning site: HindIII/BamHI
Gene length: 1360bp
The gene sequence of circSP3:
atgaccgctcccgaaaagcccgtgaaacaagaggaaatggctgccttggac
gtggatagcggcggcggtggcggcggcggcggccacggcgagtatc
tgcagcagcagcaacagcacggaaacggtgcggtggcggcggcagcggc
ggcccaggacactcagccgtcaccgctcgctctgctggccgctacctgcagc
aagatagggccgccatcgccgggcgacgacgaggaggaggcggccgcc
gcagccggggcccccgccgccgccggagcgacaggtgatttggcttctgca
cagttaggaggagcaccaaaccgatgggaggttttgtcagccacacctacaa
ctataaaagatgaagctggtaatctagtccagattccaagtgctgctacttcaag
tgggcagtatgttcttccccttcagaatttgcagaatcaacaaatattttccgttgc
accaggatcagattcatcaaatggtacagtgtccagtgttcaatatcaagtgata
ccacagatccagtcagcagatggtcagcaggttcaaattggtttcacaggctct
tcagataatgggggtataaatcaagaaagcagtcaaattcagatcattcctggc
tctaatcaaaccttacttgcctctggaacaccttctgctaacatccagaatctcat
accacagactggtcaagtccaggttcagggagttgcaattggtggttcatctttt
cctggtcaaacccaagtagttgctaatgtgcctcttggtctgccaggaaatatta
cgtttgtaccaatcaatagtgtcgatctagattctttgggactctcgggcagttct
cagacaatgactgcaggcattaatgccgacggacatttgataaacacaggac
aagctatggatagttcagacaattcagaaaggactggtgagcgggtttctcctg
atattaatgaaactaatactgatacagatttatttgtgccaacatcctcttcatcac
agttgcctgttacgatagatagtacaggtatattacaacaaaacacaaatagctt
gactacatctagtgggcaggttcattcttcagatcttcagggaaattatatccagt
cgcctgtttctgaagagacacaggcacagaatattcaggtttctacagcacag
cctgttgtacagcatctacaacttcaagagtctcagcagccaaccagtcaagc
ccaaattgtgcaaggtattacaccacagacaatccatggtgtgcaagccagtg
gtcaaaatatatcacaacaggctttgcaaaatcttcagttgcagctgaatcctgg
aaccttttaattcaggcacagacagtgacccccttctggacaggtaacttggca
aacgtttcaagtacaaggggtccagaacttgcagaatttgcaaatacagaatac
tgctgcccaacaaataactttgacgcctgttcaaaccctcacacttggtcaagtt
gcggcaggtggagccttcacttcaactccagttagtctaagcactggtcagttg
ccaaatctacaaacagttacagtgaactctatagattctgctggtatacagctac
atccaggagagaatgctgacagtcctgcagatattaggatcaaggaagaaga
acctgatcctgaagagtggcagctcagtggtgattctaccttgaataccaatga
cctaacacacttaagagtacaggtggtagatgaagaaggggaccaacaacat
caagaaggaaaaagacttcggagggtagcttgcacctgtcccaactgtaaag
aaggtggtggaagaggtaccaatcttgggaaaaagaagcaacacatttgtcat
ataccaggatgtggtaaagtctatgggaagacctcacatctgagagctcatctg
cgttggcattctggagaacgcccttttgtttgtaactggatgtactgtggtaaaag
atttactcgaagtgatgaattacagaggcacagaagaacacatacaggtgaga
agaaatttgtttgtccagaatgttcaaaacgctttatgagaagtgaccaccttgc
caaacatattaaaacacaccagaataaaaaggtattcactctagcagtacagt
gctggcatctgtggaagctgcgcgagatgatactttgattactgcaggaggaa
caacgcttatccttgcaaatattcaacaaggttctgtttcagggataggaactgtt
aatacttccgccaccagcaatcaagatatccttaccaacactgaaatacctttac
agcttgtcacagtttctggaaatgagacaatggagtaa

Supplementary File 2. The miR-198 target predicted by the online database.

VCP	CDH11	CCND2
PAK6	ADARB1	DGCR8
MSI2	SERPINF2	HMGA1
DENND2D	CDK6	PAPSS1
APBA2	DKK3	MYB
SHOX2	PTEN	HOXA1
CSF1	PER2	ARHGEF9
ELAVL1	NLK	SMAD2
CDK4	LARP1	CTBP2
POLR2E	BIRC5	FANCD2
NOVA1	SP1	FUT8
MAPRE1	DDR1	PPP2R5E
BMF	FOXJ1	NTRK3
SPRY2	MET	NCOR2
EGF	SERPINA5	BTG2
GPSM2	PAFAH1B1	SRC
SLC2A1	NEK8	FASN
SETD4	BVES	H2AFX
PCDH9	TLN1	CNR1
CACNA1	HDAC4	PEA15
OGT	FRS2	SETDB1
TNFRSF18	MSR1	PDCD1LG2
FTCD	SPTBN1	CAPN2
HDGF	PEG10	TNK2
PPP1R9B	PTPN1	HOXB13
PLAU	TNRC6B	ADAM1
RAD52	IDS	TFCP2
CD4	HFE	POLD1
TRIM3	ARHGAP1	SORT1
ITGA5	TLN2	CDKN2B
INPPL1	PPARA	YWHAG
ASPH	THBS1	MAP2K7
STK17B	CBL	HDLBP
PSD3	ZEB2	BAG3
AMFR	MLL2	TPM3
CTGF	KRT14	PVR
